# Disentangling the Association between Statins, Cholesterol, and Colorectal Cancer: A Nested Case-Control Study

**DOI:** 10.1371/journal.pmed.1002007

**Published:** 2016-04-26

**Authors:** Ronac Mamtani, James D. Lewis, Frank I. Scott, Tariq Ahmad, David S. Goldberg, Jashodeep Datta, Yu-Xiao Yang, Ben Boursi

**Affiliations:** 1 Division of Hematology/Oncology, University of Pennsylvania, Philadelphia, Pennsylvania, United States of America; 2 Center for Clinical Epidemiology and Biostatistics, University of Pennsylvania, Philadelphia, Pennsylvania, United States of America; 3 Division of Gastroenterology, University of Pennsylvania, Philadelphia, Pennsylvania, United States of America; 4 Section of Cardiovascular Medicine, Yale School of Medicine, New Haven, Connecticut, United States of America; 5 Department of Surgery, University of Pennsylvania, Philadelphia, Pennsylvania, United States of America; 6 Tel-Aviv University, Tel-Aviv, Israel; Stanford University, UNITED STATES

## Abstract

**Background:**

Several prior studies have found an association between statin use and reduced risk of colorectal cancer. We hypothesized that these findings may be due to systematic bias and examined the independent association of colorectal cancer risk with statin use, serum cholesterol, and change in cholesterol concentration.

**Methods and Findings:**

22,163 colorectal cancer cases and 86,538 matched controls between 1995 and 2013 were identified within The Health Improvement Network (THIN) a population-representative database. Conditional logistic regression models estimated colorectal cancer risk with statin use, serum total cholesterol (mmol/L), and change in total cholesterol level. We confirmed a decreased risk of colorectal cancer with statin use (long-term: odds ratio [OR], 0.95; 95% confidence interval [CI], 0.91–0.99; short-term: OR, 0.92; 95% CI, 0.85–0.99). However, to assess whether the observed association may result from indication bias, a subgroup analysis was conducted among patients prescribed a statin. In this subgroup (*n* = 5,102 cases, *n* = 19,032 controls), 3.1% of case subjects and 3.1% of controls discontinued therapy. The risk of colorectal cancer was not significantly different among those who continued statin therapy and those who discontinued (OR, 0.98; 95% CI, 0.79–1.22). Increased serum cholesterol was independently associated with decreased risk of colorectal cancer (OR, 0.89 per mmol/L increase; 95% CI, 0.87–0.91); the association was only present if serum cholesterol was measured near the cancer diagnosis (<6 mo: OR, 0.76; 95% CI, 0.47–0.61; >24 mo: OR, 0.98; 95% CI, 0.93–1.03). Decreases in serum total cholesterol >1 mmol/L ≥1 year prior to cancer diagnosis were associated with subsequent colorectal cancer (statin users: OR, 1.25; 95 CI%, 1.03–1.53; nonusers: OR, 2.36; 95 CI%, 1.78–3.12). As an observational study, limitations included incomplete data and residual confounding.

**Conclusions:**

Although the risk of colorectal cancer was lower in statin users versus nonusers, no difference was observed among those who continued versus discontinued statin therapy, suggesting the potential for indication bias. The association between decreased serum cholesterol and colorectal cancer risk suggests a cholesterol-lowering effect of undiagnosed malignancy. Clinical judgment should be used when considering causes of cholesterol reduction in patients, including those on statin therapy.

## Abstract 2

In a nested case-control study, Ronac Mamtani and colleagues investigate the associations between cholesterol, statin use, and colorectal cancer among primary care patients in the United Kingdom.

## Author Summary

**Background:**

- In the United States and United Kingdom, cholesterol monitoring is recommended for patients aged 40 years or older, and statins are widely used to treat high cholesterol.
- In addition to preventing cardiovascular disease, statins might also potentially have cancer prevention effects.
- A number of studies have shown that compared to people not using statins, those receiving statins appear to have a lower colon cancer risk.

**Why Was This Study Done?:**

- However, the prior studies that found that statins reduce colon cancer risk did not account for the effect of blood cholesterol level on cancer risk.
- If high blood cholesterol (the indication for statins) lowers cancer risk, then the previously reported associations between statins and cancer risk could be biased toward an artificially protective effect of statins (indication bias).
- Our study was designed specifically to elucidate the reasons behind the observed association between statin therapy and reduced colon cancer risk.

**What Did the Researchers Do and Find?:**

- We performed a case-control study using a computerized database of electronic records from >10 million UK patients in primary care practices (The Health Improvement Network [THIN]).
- We identified 22,163 patients with colon cancer (cases) and 86,538 patients without colon cancer (controls), comparing statin use and blood cholesterol level between the two groups.
- We confirmed a decreased risk of colon cancer with statin use compared to no use.
- After accounting for indication bias by comparing statin-treated patients to those who discontinued statins, we no longer observed a difference in the risk of colorectal cancer.
- Rather, higher blood cholesterol level was associated with lower colon cancer risk and lower blood cholesterol level with higher colon cancer risk. For example, decreases in total cholesterol by >1 mmol/L (~38.6 mg/dl) at least a year before the cancer diagnosis were associated with 1.25-fold and 2.36-fold increased risk of colorectal cancer in users and nonusers of statin.

**What Do These Findings Mean?:**

- Statins should not be prescribed for the purpose of colon cancer prevention.
- Unexplained decreases in blood total cholesterol should alert physicians to consider colon cancer as one potential explanation.

## Introduction

Statins are cholesterol-lowering medications indicated for the prevention of cardiovascular events and used in up to 25% of adults in the United States and United Kingdom [1,2]. Under new US and UK guidelines for the management of cholesterol [3,4], it is estimated that millions more adults would be eligible for statin therapy including those with low cardiovascular risk [5]. These recommendations have been surrounded by controversy regarding the risks and benefits of statin therapy. Safety concerns with statins include myopathy, cognitive impairment, weight gain, and diabetes mellitus [6].

A potential added benefit of statin therapy is reduction in cancer incidence, particularly colorectal cancer. Although meta-analyses of randomized controlled trials have shown no effect of statins on overall cancer incidence [7,8], a meta-analysis of observational studies found a modest but statistically significant reduction in colorectal cancer risk with statin therapy (relative risk [RR]: 0.89, 95% confidence interval [CI] 0.84–0.95) [9]. It is unclear from these studies whether it is statin use or the hyperlipidemia that prompted statin use, that may be associated with colorectal cancer. Confounding by indication, or indication bias, is a common bias in observational studies and occurs when the indication (high cholesterol) for the medication under study (statin) is also associated with the outcome of interest (colon cancer) [10]. Indeed, some studies have observed that higher serum cholesterol, the indication for statin therapy, lowers colorectal cancer risk [11–13]. However, to our knowledge, prior studies that led to the conclusion that statin treatment reduces colorectal cancer risk did not account for the potential confounding effects of pre-treatment serum cholesterol. Furthermore, there are few data on the relation between the time of cholesterol measurement and the diagnosis of colorectal cancer [11], and there are no data on change in serum cholesterol and the risk of colorectal cancer.

To distinguish the putative effect of statin therapy from the underlying indication for therapy (hyperlipidemia), we conducted a nested case-control study in which we compared statin continuers with discontinuers and examined associations of levels of serum total cholesterol, time of cholesterol measurement, and change in cholesterol with colorectal cancer risk.

## Methods

### Data Source

We used data from The Health Improvement Network (THIN), a primary care medical records database representative of the broader United Kingdom (http://www.epic-uk.org/our-data/our-data.shtml). THIN contains de-identified electronic records of over 12 million patients, of which 3.6 million patients are actively registered in over 550 general practices in the UK. Data available in THIN include demographic information, medical diagnoses, drug prescriptions, lifestyle characteristics such as smoking and alcohol consumption, clinical measurements recorded by general practitioners (GPs) such as height and weight, and laboratory values including serum cholesterol. Medical diagnoses are recorded using Read codes, the standard primary care classification system in the UK [14]. Data quality is monitored through routine analysis and auditing of the entered data [15]. Average follow-up time exceeds 5 y per patient. The accuracy and completeness of THIN data is well documented and the database has been previously used to study the pharmacoepidemiology of colorectal cancer [16,17]. The positive predictive value of a colorectal cancer diagnosis recorded in THIN has been shown to be high (>95%) [18].

### Study Design and Population

We conducted a nested case-control analysis within THIN, examining the associations of colorectal cancer with statin use, serum total cholesterol, and change in total cholesterol level. Patients who registered with a general practitioner within THIN from 1995 to 2013 were eligible for inclusion. Follow-up started at the later of either the date the practice started using the electronic medical record (Vision software) or the date the patient registered with their general practitioner, and ended on the index date (described below).

### Definition of Cases and Controls

Cases were identified as those patients with at least one diagnostic code for colorectal cancer (S1 Text) during follow-up and aged more than 40 y at the time of colorectal cancer diagnosis. Subjects with a diagnosis of colorectal cancer within the first 6 mo of the follow-up period were excluded to avoid misclassification of prevalent colorectal cancer as incident colorectal cancer [19].

Selection of controls was based on incidence density sampling [20]. In this method, potential controls included those who were at risk for colorectal cancer at the time that the matched case was diagnosed with colorectal cancer (S1 Fig). Thus, for each individual with colorectal cancer, up to four controls who were alive and without colorectal cancer at the time of the patient’s diagnosis were randomly selected after matching on age (±5 y), sex, practice site, duration of follow-up, and calendar period. The date that the case subject was first diagnosed with colorectal cancer served as the index date for both the case subject and for the matched control. The incidence density sampling design assures equal time during which case and control subjects are at risk for the exposures of interest and generates odds ratios (ORs) interpretable as unbiased estimates of incidence rate ratios [21].

### Exposures

In the statin analysis, the primary exposure variable was statin use. In an analysis of statin users versus nonusers, consistent with prior studies, statin use was categorized as never use, short-term use (first prescription date <1 year before index date and last prescription date <6 mo before index date), or long-term use (first prescription date >1 year before index date and last prescription date <6 mo before index date). In the statin continuer versus discontinuer analysis, statin continuers were defined identically to long-term users (i.e., receipt of least two prescriptions, first prescription >1 year before index date and last prescription <6 mo before index date). Statin discontinuers were defined as having received a single prescription >1 year before the index date.

In the hyperlipidemia analysis, the primary exposure variable was serum total cholesterol (mmol per liter), using values measured at different time-windows prior to the index date. The components of total cholesterol measurements were considered secondary exposure variables.

### Statistical Analyses

#### Association between statin therapy and colorectal cancer

To assess the association of statin therapy on colorectal cancer risk before and after accounting for confounding by indication, separate conditional logistic regression models were used to estimate odds ratios (ORs) and 95% confidence interval (CI) for colorectal cancer. The first model compared statin users with nonusers. The second model, to account for confounding by indication, compared statin continuers with discontinuers and adjusted for pre-treatment total cholesterol (last recorded cholesterol value prior to statin initiation) among the subgroup of patients prescribed a statin. Each model was conditioned upon the five matching factors and adjusted a priori for variables known to influence colorectal cancer risk, including obesity (body mass index [BMI] > 30 kg/m^2^), smoking status (ever versus never), diabetes mellitus, alcohol consumption, prior bowel screening, and use of aspirin or non-steroidal anti-inflammatory drugs, hormone replacement therapy, or non-statin cholesterol-lowering medication. All covariates were measured before the index date.

#### Association between hyperlipidemia and colorectal cancer

Conditional logistic regression was also used to estimate adjusted ORs and 95% CIs for colorectal cancer risk with hyperlipidemia (categorized as total cholesterol levels of <4, 4–5, 5–6, 6–7, and >7 mmol/L), independent of statin use. This analysis only included statin nonusers; the reference group was a total cholesterol level of <4 mmol/L, consistent with UK guidelines for desirable total cholesterol level [22]. Cholesterol was also modeled as a continuous variable. To assess whether the association of hyperlipidemia on colorectal cancer risk is influenced by time of cholesterol measurement, we tested for an interaction between serum cholesterol and time of measurement and reported adjusted ORs and 95% CIs for colorectal cancer risk stratified by different time-windows (<6 mo, 6–12, 12–24, and >24) before a cancer diagnosis.

#### Association between reduction in serum cholesterol and colorectal cancer

Finally, to assess the association between change in serum total cholesterol level and colorectal cancer risk, adjusted ORs for colorectal risk were calculated per-unit (mmol/L) decrease in total cholesterol (modeled as a continuous variable) and for <1 unit and >1 unit decreases in total cholesterol (modeled as a categorical variable). Analyses were conducted separately for statin users and nonusers and included subjects with at least two total cholesterol measurements, separated by at least 1 y, with the last measurement occurring at least 1 y before the date of colorectal cancer diagnosis. The reference group for the categorical analysis included subjects with no change or increases in total cholesterol between the measurements. Conditional logistic regression models were adjusted for the pre-specified set of confounders, and the following additional potential confounders: use of non-statin cholesterol-lowering medications, weight loss during follow-up, and the first available total cholesterol level recorded.

STATA version 13.0 was used for statistical analyses (StataCorp, CollegeStation, TX, US). All statistical tests were two-sided. All primary analyses were specified a priori. The study protocol was approved by the University of Pennsylvania’s Institutional Review Board and the UK’s Scientific Review Committee.

## Results

Table 1 displays characteristics of 22,163 subjects with colorectal cancer and 86,538 matched controls. The median duration from start of follow-up to a diagnosis of colorectal cancer was 6 y. Case subjects were more likely than controls to have a history of smoking, diabetes, obesity, and alcohol use. Statins were continued in 4,946 (22.3%) cases and 18,433 (21.3%) controls, and discontinued in 156 (0.70%) cases and 599 (0.69%) controls. The median total cholesterol level was 5.5 mmol/L (interquartile range [IQR] 4.8–6.3) in cases and 5.7 mmol/L (IQR 5.0–6.4) in controls.

**Table 1:** Characteristics of colorectal cancer cases and control subjects.

	Cases (*n* = 22,163)	Controls (*n* = 86,538)
Age at index date, median, y (IQR)	72.3 (63.8–79.6)	72.0 (63.4–79.3)
Male sex (%)	12,190 (55.0)	47,602 (55.0)
Duration of follow-up, median, y (IQR)^a^	6.0 (3.0–9.0)	6.0 (3.0–9.0)
Cigarette smoking history (%)		
Never	11,789 (53.2)	50,737 (58.6)
Ever	10,374 (46.8)	35,801 (41.4)
Diabetes mellitus (%)	2,859 (12.9)	8,622 (10.0)
Obesity (BMI ≥ 30 kg/m^2^) (%)	4,593 (20.7)	15,899 (18.4)
Prior cancer^b^	972 (4.4)	2,712 (3.1)
Alcohol use (%)^c^	11,514 (52.0)	42,088 (48.6)
Performance of bowel screening (%)^d^	1,029 (4.8)	3,822 (4.7)
Hormone replacement therapy (%)	1,396 (6.3)	5,788 (6.7)
Chronic NSAID or ASA use (%)^e^	678 (3.1)	2,738 (3.2)
Non-statin cholesterol-lowering medication (%)	446 (2.0)	1,958 (2.3)
Statin use (%)		
Continuer^f^	4,946 (22.3)	18,433 (21.3)
Discontinuer^g^	156 (0.70)	599 (0.69)
Total cholesterol level, mmol/L^h^	5.5 (4.8–6.3)	5.7 (5.0–6.4)

SD, standard deviation; y, years; NSAID, non-steroidal anti-inflammatory drugs; IQR, interquartile range.

^a^ Before index date

^b^ Diagnosis of lung, prostate, or breast cancer before index date

^c^ Any use

^d^ Colonoscopy, sigmoidoscopy, or fecal occult blood screening >2 y prior to index date

^e^ Cumulative duration of therapy more than 5 y

^f^ Receipt of two or more prescriptions, first prescription >1 y before index date and last prescription within 6 mo before index date

^g^ Receipt of 1 prescription >1 y before index date

^h^ Last available total cholesterol value prior to statin initiation, before index date

### Association between Statin Therapy and Colorectal Cancer

As shown in Table 2, statin use was associated with a statistically significantly decreased risk of colorectal cancer with both short-term (OR 0.92, 95% CI 0.85–0.99) and long-term use (OR 0.95, 95% CI 0.91–0.99) relative to no use. However, after accounting for confounding by indication by comparing statin continuers with discontinuers and adjusting for pre-treatment total cholesterol, this association was no longer noted (OR 0.98, 95% 0.79–1.22) (Table 3).

**Table 2:** ORs for colorectal cancer risk in statin users relative to nonusers.

Covariate	Cases *n* = 21,247 (%)	Controls *n* = 80,219 (%)	Adjusted OR^a^ (95% CI)
**Statin use**			
Never	15,407 (72.5)	59,501 (74.2)	Reference
Short-term	904 (4.3)	3,406 (4.2)	0.92 (0.85–0.99)
Long-term	4,936 (23.2)	17,312 (21.6)	0.95 (0.91–0.99)

Short-term User, first prescription date less than 1 y before index date, and last prescription date less than 6 mo before index date; Long-term User, first prescription date more than 1 y before index date, and last prescription date less than 6 mo before index date

^a^ Adjusted for obesity (BMI ≥ 30 kg/m^2^), ever smoking, chronic use of aspirin/NSAIDs (>5 y), hormone replacement therapy, alcohol consumption, diabetes mellitus, performance of bowel screening, and use of non-statin cholesterol-lowering medication.

**Table 3:** ORs for colorectal cancer risk in statin continuers relative to discontinuers, adjusting for pre-treatment total cholesterol.

Covariate	Cases *n* = 22,163 (%)	Controls *n* = 86,538 (%)	Adjusted OR^a^ (95% CI)	Most Fully Adjusted OR^b^ (95% CI)
**Statin use**				
Discontinuers	156 (0.70)	599 (0.69)	Reference	Reference
Continuers	4,946 (22.3)	18,433 (21.3)	0.98 (0.82–1.17)	0.98 (0.79–1.22)
**Pre-treatment Total Cholesterol**				
<4 mmol/L	760 (6.8)	1,758 (4.3)	-	Reference
4–5 mmol/L	2,549 (22.8)	7,864 (19.2)	-	0.75 (0.67–0.84)
5–6 mmol/L	3,881 (34.8)	14,986 (36.6)	-	0.59 (0.53–0.66)
6–7 mmol/L	2,704 (24.2)	10,840 (26.5)	-	0.59 (0.52–0.66)
>7 mmol/L	1,274 (11.4)	5,494 (13.4)	-	0.53 (0.47–0.61)
Continuous^c^	11,168 (50.4)	40,942 (47.3)	-	0.89 (0.87–0.91)

Statin Continuer, receipt of two or more prescriptions, first prescription >1 y before index date and last prescription within 6 mo before index date; Statin Discontinuer, receipt of one prescription >1 y before index date.

^a^ Adjusted for obesity (BMI ≥ 30 kg/m^2^), ever smoking, chronic use of aspirin/NSAIDs (>5 y), hormone replacement therapy, alcohol consumption, diabetes mellitus, performance of bowel screening, and use of non-statin cholesterol-lowering medication.

^b^ Adjusted for variables above, and pre-treatment cholesterol measured as the last available total cholesterol level before statin initiation in statin continuers and statin discontinuers.

^c^ Per 1 unit (mmol/L) increase in serum total cholesterol

### Association between Hyperlipidemia and Colorectal Cancer

**Fig 1:**
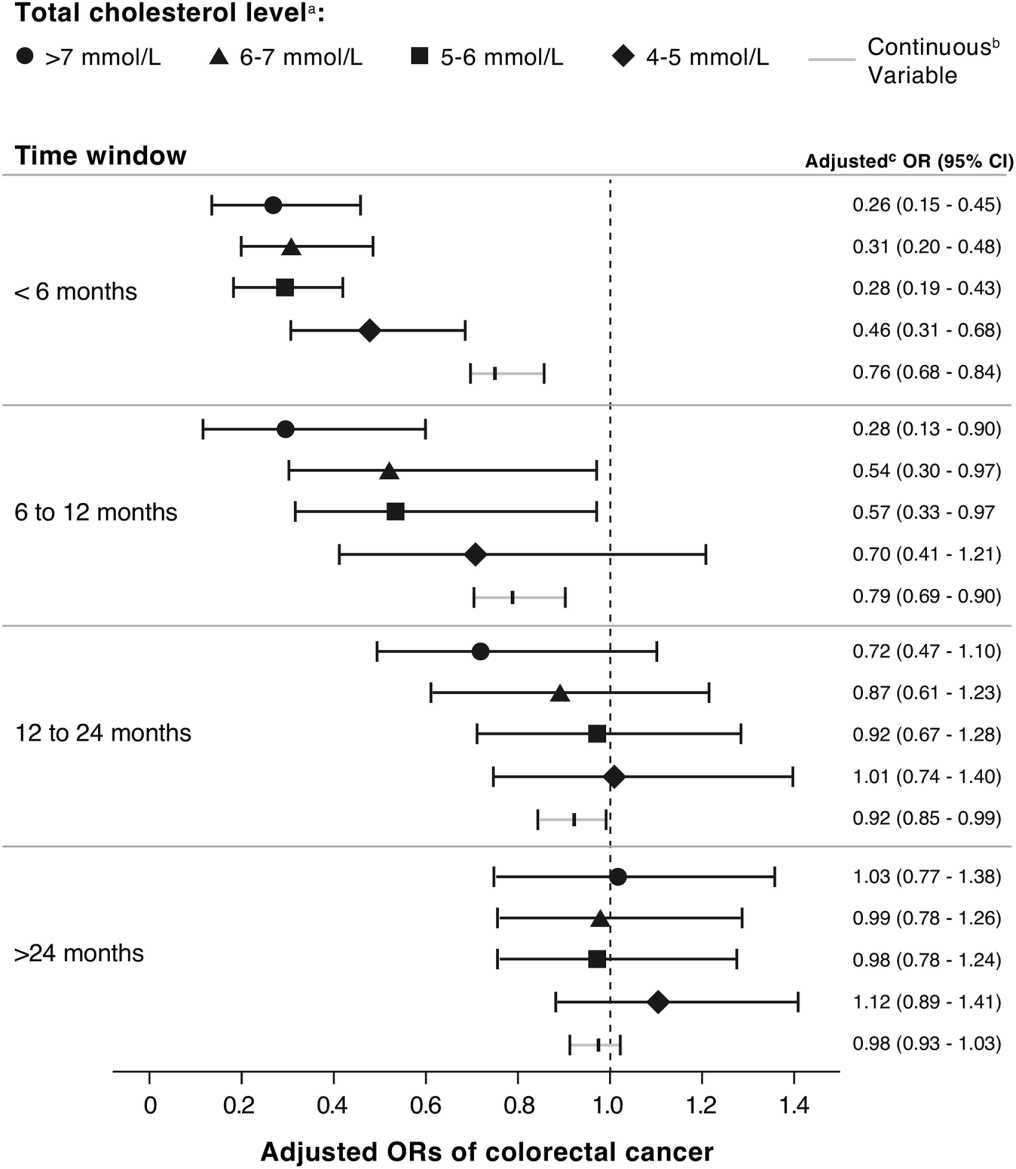
ORs for association between colorectal cancer risk and total cholesterol measured at different time intervals before colorectal cancer diagnosis, among statin nonusers (*n* = 15,052 cases; *n* = 46,043 controls). ^a^ Last total cholesterol value measured in each specified time window prior to the index date of colorectal cancer diagnosis. ^b^ Adjusted for obesity (BMI ≥ 30 kg/m^2^), ever smoking, chronic use of aspirin or NSAIDs, hormone replacement therapy, alcohol consumption, diabetes mellitus, performance of bowel screening, and non-statin cholesterol-lowering medication. ^c^ Per 1 mmol/L increase in serum total cholesterol.

We found a statistically significantly decreased risk of colorectal cancer with increasing serum total cholesterol (OR 0.89 per unit [mmol/L] increase, 95% CI 0.87–0.91) (Table 3). When examined as categorical, the OR for the highest (>7 mmol/L) versus lowest (<4 mmol/L) category of total cholesterol was 0.53 (95% CI 0.47–0.61). To further explore the association between elevated serum cholesterol and colorectal cancer risk independent of statin use, we calculated the risk of colorectal cancer risk among statin nonusers at different time intervals of cholesterol measurement before the cancer diagnosis (Fig 1). There was a statistically significant interaction between serum cholesterol and time of measurement (*p* = 0.007). The association between elevated total cholesterol level and decreased colorectal cancer risk was stronger if serum cholesterol was measured near the cancer diagnosis (<6 mo before diagnosis: OR 0.76, 95% CI 0.68–0.84; >24 mo before diagnosis: OR 0.98, 95% CI 0.93–1.03).

### Association between Reduction in Serum Cholesterol and Colorectal Cancer

Lastly, we performed additional analyses to determine whether subjects with decreasing total cholesterol had an increased risk of colorectal cancer (Table 4). These analyses were restricted to subjects with at least two total cholesterol measurements, separated by at least 1 y, with the last measurement occurring at least 1 y before the colorectal cancer diagnosis. After adjusting for use of non-statin cholesterol-lowering medications, baseline total cholesterol level, and weight loss during follow-up, as well as the a priori variables, the risk of colorectal cancer in statin nonusers was statistically significantly increased for a 1 mmol/L decrease in serum total cholesterol (OR 1.49 [1.32–1.69]) and even higher for a >1 mmol/L decrease (OR 2.36 [1.78–3.12). Similar analyses among statin users produced lower ORs (OR 1.23 per 1 mmol/L decrease, 95 CI% 1.15–1.32; OR 1.25 for >1 mmol/L decrease, 95 CI% 1.03–1.53). Additionally, cases with colorectal cancer had statistically significantly greater mean reductions in total cholesterol among statin users and nonusers (1.54 and 0.34 mmol/L, respectively) compared to controls (1.44 and 0.14 mmol/L, respectively) [*p* < 0.002 for both comparisons].

**Table 4:** ORs for colorectal cancer risk by change in serum total cholesterol.

Model	Cases^a^		Controls^a^	OR (95% CI) for no change or increase^b^	OR (95% CI) for <1 mmol/L decrease	OR (95% CI) for >1 mmol/L decrease	OR (95% CI) per 1 mmol/L decrease
Statin nonusers							
Adjusted^c^		1,901	2,706	1.00	1.29 (1.12–1.48)	2.37 (1.95–2.89)	1.47 (1.35–1.60)
Most fully adjusted^d^		1,184	1,599	1.00	1.28 (1.06–1.54)	2.36 (1.78–3.12)	1.49 (1.32–1.69)
Statin users							
Adjusted^c^		3,202	5,624	1.00	1.21 (1.03–1.43)	1.25 (1.10–1.45)	1.09 (1.04–1.13)
Most fully adjusted^d^		2,525	4,255	1.00	1.16 (0.96–1.40)	1.25 (1.03–1.53)	1.23 (1.15–1.32)

^a^ Limited to cases and controls with at least two total cholesterol measurements, separated by at least 1 y, with the last measurement occurring at least 1 y before the index date of colorectal cancer diagnosis.

^b^ Reference group includes subjects with no change or increase in total cholesterol between the first and last total cholesterol measurement recorded

^c^ Adjusted for age, sex, duration of follow-up, calendar period, obesity (BMI ≥ 30 kg/m2), ever smoking, chronic use of aspirin or non-steroidal anti-inflammatory medications, hormone replacement therapy, alcohol consumption, diabetes mellitus, and performance of bowel screening

^d^ Adjusted for variables in adjusted model, as well as non-statin cholesterol-lowering medication, weight loss during follow-up, and first available total cholesterol measurement during follow-up

### Sensitivity Analyses

Following peer review, several sensitivity analyses were conducted. To assess the impact of missing BMI data, we used linear regression to impute missing BMI values; estimates were largely unchanged (S1 Table). To assess whether the results might have been biased by a history of other common cancers prior to colorectal cancer, we excluded subjects with prior lung, prostate, and breast cancers (S1 and S2 Tables); results were similar to the primary analysis. To test whether the association of colorectal cancer with hyperlipidemia differed by statin use, analyses were conducted in both statin nonusers and users (S3 Table). Similar patterns of an inverse association between cancer risk and serum cholesterol were observed, regardless of statin use. To examine whether the association of colorectal cancer risk differed by lipid profile component, analyses of cancer risk with reduction in triglycerides (TG), high-density lipoprotein cholesterol (HDL), and low-density lipoprotein cholesterol (LDL) were performed. Significant increases in risk were seen with reductions in LDL (OR 1.37, 95% CI 1.11–1.69) and HDL (OR 2.14, 95% CI 1.49–3.07), and less so with TG (1.16, 1.06–1.27) (S4 Table).

## Discussion

**Fig 2:**
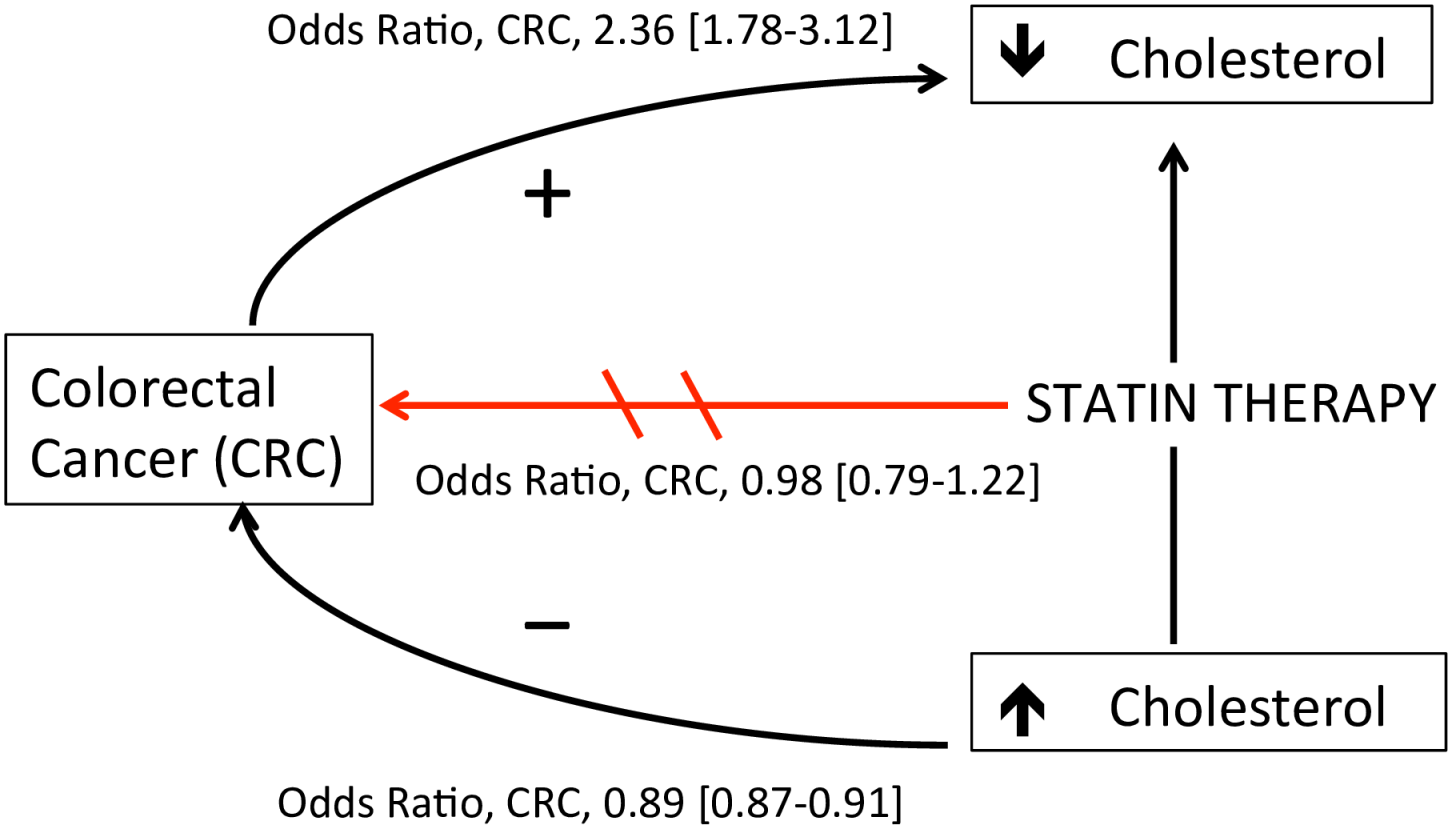
Proposed model of the association between statins, cholesterol, and colorectal cancer (CRC) risk. Statin therapy was not associated with decreased CRC risk after accounting for cholesterol level (OR 0.98, 95% CI 0.79–1.22). Increased serum cholesterol was independently associated with decreased CRC risk (OR 0.89 per 1 mmol/L increase, 95% CI 0.87–0.91). Decreases in serum cholesterol >1 mmol per liter ≥1 y before cancer diagnosis was associated with increased CRC risk in statin users (OR 1.25, 95% CI 1.03–1.53) and nonusers (OR 2.36, 95% CI 1.78–3.12), suggesting a cholesterol-lowering effect of undiagnosed malignancy. Arrow directions are based on the authors’ interpretation.

This case-control analysis was conducted to clarify the independent association of colorectal cancer risk with statin use, serum total cholesterol, and change in total cholesterol level. When comparing statin-treated patients to those who discontinued therapy after a single prescription, statin therapy was not associated with decreased colorectal cancer risk. Rather, serum cholesterol was inversely related to short-term colorectal cancer risk regardless of statin use: decreases in serum total cholesterol by more than 1 mmol/L occurring at least a year before the cancer diagnosis were associated with a 1.25-fold and 2.36-fold increased risk of colorectal cancer in users and nonusers of statin therapy, respectively. Together, these data demonstrate a complex association between statins, cholesterol, and colorectal cancer (Fig 2), suggesting that unexplained cholesterol lowering in statin users or nonusers may be a marker of undiagnosed colorectal cancer.

A number of observational studies have reported a decreased risk of colon cancer with statin therapy [9]. To our knowledge, all prior studies examining the risk of colorectal cancer among patients using statins did not account for confounding by indication, and thus could be biased toward a protective association of statin therapy if hyperlipidemia is associated with lower colorectal cancer risk. Statistical methods used to reduce confounding by indication, such as propensity scores adjustment and the instrumental variable analysis, do not completely resolve this bias [23]. Other methods, such as comparing users of one drug to users of a comparable drug for the same indication [24], improve comparability between exposures but may introduce other biases shown to result in artificial reductions in cancer risk observed with a drug [25]. Recognizing that serum cholesterol level is not the only factor that drives statin use, we compared statin continuers to discontinuers, such that each group had sufficient indication that statins were prescribed. Furthermore, unlike most prior studies, we controlled for differences in baseline (pre-treatment) serum cholesterol level between statin continuers and discontinuers that might have influenced colorectal cancer risk. Using these methods, our results are consistent with that from post hoc analyses of randomized controlled trials, suggesting that statin use does not decrease the risk of colorectal cancer [26].

Our finding that high pre-treatment serum cholesterol was associated with decreased risk of colorectal cancer could be due to chance, causation, or reverse causation. Chance is unlikely given the presence of a dose response (11% decreased risk per mmol/L increase in cholesterol) and that the inverse association was observed in earlier studies pre-dating the marketing of statins [11]. A causal association is possible, but data supporting biological plausibility are limited to one study showing improved host antitumor immunity in individuals with hyperlipidemia relative to hypolipidemia [27]. Reverse causality is the most likely explanation, as accelerated lipid metabolism and cholesterol-lowering has been suggested as an early manifestation of colon cancer [28]. This interpretation is supported by our observation that the inverse association between serum cholesterol and colon cancer risk was attenuated as the time interval between cholesterol measurement and cancer diagnosis increased from <6 mo (OR 0.76, 95% CI 0.68–0.84) to >24 mo (OR 0.98, 95% CI 0.93–1.03).

Another novel aspect of this study was the evaluation of colorectal cancer risk with change in serum total cholesterol levels. This analysis, which adjusted for weight loss and key colorectal cancer risk factors and used serum cholesterol values measured ≥1 y before the cancer diagnosis, found a 2.36-fold increased risk of colorectal cancer with serum cholesterol reductions of more than 1 mmol/L (39 mg per deciliter) in statin nonusers and a 1.25-fold increased risk in statin users. Given the rapid onset of cholesterol lowering by statin therapy and the relatively long latency period required for colorectal carcinogenesis, we believe the modestly increased cancer risk associated with reduction in serum cholesterol concentration observed in statin users was related to cholesterol lowering from undiagnosed malignancy and not a causal association with statin therapy. To our knowledge, the only other study to examine the association between reduction in serum cholesterol and cancer risk was a meta-analysis of statin clinical trials, which combined all cancers, had shorter follow-up, and lacked patient-level data [29]. Combining all cancers risks bias towards the null if the effect is not universal across all cancers.

Results from this study may have important implications for clinicians. Our data suggest that an otherwise unexplained reduction in serum total cholesterol by more than 1 mmol/L (38.6 mg/dL) may not be a favorable indicator, but rather a signal of occult colorectal cancer. The increase in risk of colorectal cancer, about 2.4-fold, is similar to the relative risk increase among individuals with a family history of colorectal cancer, in whom earlier screening is recommended [30]. Thus, this study highlights the possibility of using total cholesterol as a blood biomarker for the risk or early detection of colorectal cancer. Regardless, in all adults, primary care clinicians should perform an individualized risk assessment of colorectal cancer and adhere to recommended screening guidelines [31]. Indeed, tumors detected by screening may be of early stage and therefore curable [32].

Cholesterol-lowering is a common treatment strategy for the management of patients at risk for coronary heart disease. New cholesterol guidelines expanding the indications for statin therapy are expected to result in an increased use of statins [33,34]. Thus, our data may also provide important information to patients and clinicians indicating that statin therapy does not appear to be associated with reduced colorectal cancer risk, but unexplained reductions in serum total cholesterol should alert physicians to consider colorectal cancer as one potential explanation.

This study has several limitations. Serum cholesterol was categorized into clinically relevant cut-points according to UK practice guidelines. While categorization avoids assumptions of linearity, it reduces statistical power and may conceal non-linear relationships between total cholesterol and colorectal cancer [35]. Because regression models met the assumption of linearity (S2 Fig), both continuous (per unit change) and categorical (by cholesterol level) analyses were presented.

The study population had incomplete data on BMI. Subjects with missing BMI were categorized as non-obese. Under this assumption, the obesity prevalence in our study (18%–21%) was similar to that reported by the Health Survey for England in 2013 (23%–26%) [36]. Furthermore, a sensitivity analysis using multiple imputation for missing BMI produced similar results to the primary analysis.

The study relied on prescriptions written by general practitioners and may not reflect actual drug dispensing or use. However, the majority of recorded prescriptions in THIN are dispensed (~97%), as shown in one recent study of the UK National Health System [37]. Although the median duration of the study was 6 y, colonic tumorigenesis generally occurs over a long period of time [38]. Thus, additional follow-up is required to evaluate longer-term effects of statin therapy on cancer risk. Even in our large cohort, the proportion of subjects discontinuing statins was small. This may have resulted in reduced statistical power for the analysis comparing colorectal cancer risk in statin-treated patients to those who discontinued therapy. We were unable to control for residual confounding by physical activity and dietary patterns; these variables may influence the risk of colorectal cancer and cholesterol level [39,40]. Finally, we were unable to test whether the association between change in serum cholesterol and colorectal cancer risk differed by cancer stage at diagnosis. If cholesterol lowering is a consequence rather than cause of colorectal cancer, then one could expect a weaker association for case subjects presenting with early- versus late-stage colorectal cancer.

In summary, although the risk of colorectal cancer was lower in statin users versus nonusers, no difference was observed among those who continued versus discontinued statin therapy, suggesting the potential for indication bias. We also demonstrate that increased serum total cholesterol is not a marker of long-term reduced risk of colorectal cancer; however, reduction in serum total cholesterol is a marker of short-term increased risk. The increased risk of colorectal cancer diagnosis in subjects with a reduction in serum total cholesterol >1 mmol/L suggests a cholesterol-lowering effect from undiagnosed malignancy. Clinical judgment should be used when considering causes of cholesterol reduction in patients, including those on statin therapy. Further studies are needed to assess the clinical utility of serum total cholesterol as a biomarker for risk or early detection of colorectal cancer.

## Supporting Information

S1 Fig Incidence density sampling design. (PDF)

S2 Fig Linear relationship of total cholesterol (per unit increase) to binary outcome of colorectal cancer (*lincheck* command, STATA). (PDF)

S1 Table ORs for colorectal cancer risk in statin continuers relative to discontinuers, using multiple imputation for missing BMI and after excluding other prior cancers. (DOCX)

S2 Table ORs for colorectal cancer risk by change in serum total cholesterol, after excluding other prior cancers. (DOCX)

S3 Table ORs for association between colorectal cancer risk and cholesterol measured at different time intervals before diagnosis among statin users, nonusers, and both. (DOCX)

S4 Table ORs for colorectal cancer risk by change in triglycerides, LDL-, and HDL-cholesterol, after excluding other prior cancers. (DOCX)

S1 Text THIN code list. (DOCX)

S2 Text STROBE checklist. (PDF)

## Abbreviations

BMI: body mass index
CI: confidence interval
CRC: colorectal cancer
GP: general practitioner
HDL: high-density lipoprotein cholesterol
IQR: interquartile range
LDL: low-density lipoprotein cholesterol
NSAID: non-steroidal anti-inflammatory drugs
OR: odds ratio
RR: relative risk
SD: standard deviation
TG: triglycerides
THIN: The Health Improvement Network
