# Supplementary figures and images for: Disentangling the Association between Statins, Cholesterol, and Colorectal Cancer: A Nested Case-Control Study
Source: PLoS Med. 2016 Apr 26;13(4):e1002007. doi: 10.1371/journal.pmed.1002007 (PMC4846028; doi:10.1371/journal.pmed.1002007)

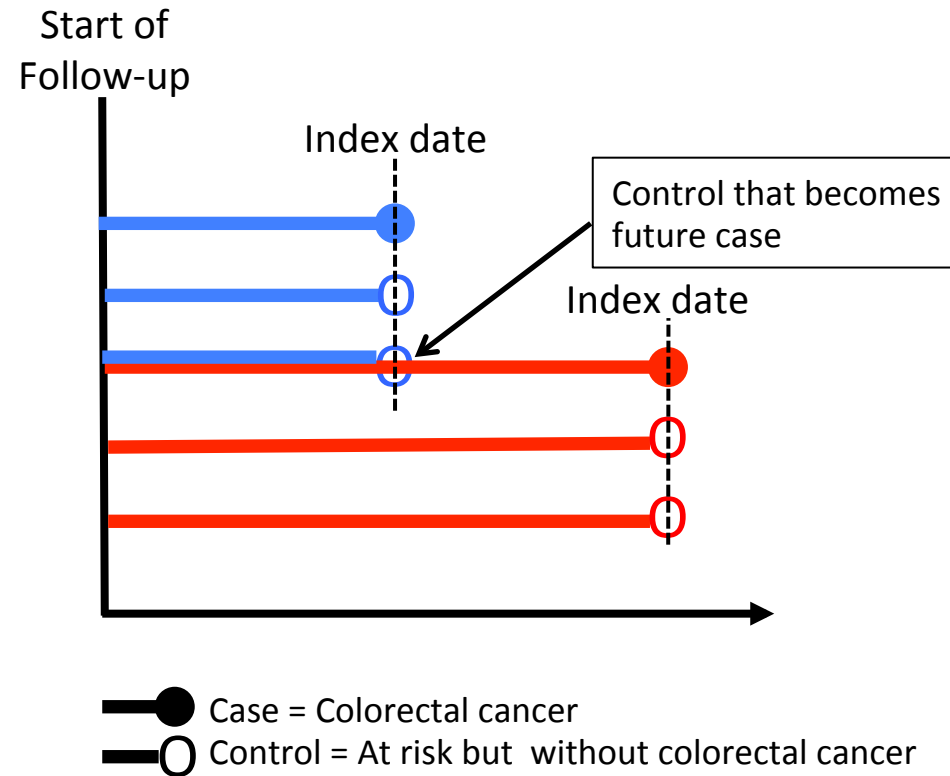

Supplement: S1 Fig — (PDF) [file pmed.1002007.s001.pdf]

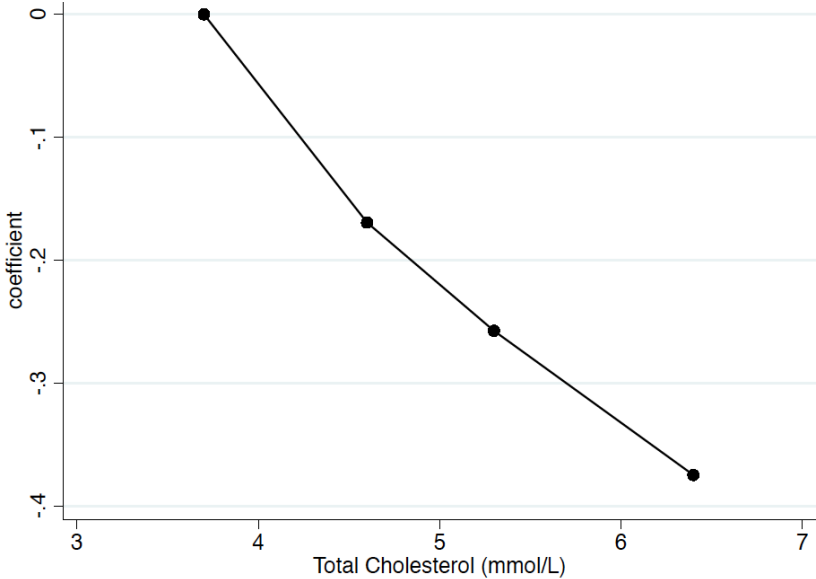

Supplement: S2 Fig — (PDF) [file pmed.1002007.s002.pdf]
