# Supplementary material for: Disentangling the Association between Statins, Cholesterol, and Colorectal Cancer: A Nested Case-Control Study
Source: PLoS Med. 2016 Apr 26;13(4):e1002007. doi: 10.1371/journal.pmed.1002007 (PMC4846028; doi:10.1371/journal.pmed.1002007)
Supplement: S1 Table — (DOCX) [file pmed.1002007.s003.docx]

| S1 Table. ORs for colorectal cancer risk in statin continuers relative to discontinuers, using multiple imputation for missing BMI, and excluding other prior cancers. | | | |
| --- | --- | --- | --- |
| **Covariate** | Original,  Adjusted OR^a^ | Multiple imputation for BMI,  Adjusted OR^b^ | Excluding prior cancers,  Adjusted OR^c^ |
| **Statin use** |  |  |  |
| Discontinuers | Reference | Reference | Reference |
| Continuers | 0.98 (0.79-1.22) | 1.00 (0.80-1.24) | 0.94 (0.75-1.18) |
| **Pre-treatment Total Cholesterol** |  |  |  |
| <4 mmol/L | Reference | Reference | Reference |
| 4-5 mmol/L | .75 (0.67 -0.84) | 0.75 (0.67 -0.84) | 0.75 (0.67-0.85) |
| 5-6 mmol/L | 0.59 (0.53-0.66) | 0.59 (0.53-0.66) | 0.60 (0.54-0.68) |
| 6-7 mmol/L | 0.59 (0.52-0.66) | 0.59 (0.52-0.66) | 0.59 (0.53-0.67) |
| >7 mmol/L | 0.53 (0.47-0.61) | 0.53 (0.47-0.60) | 0.54 (0.48-0.62) |
| Continuous ^d^ | 0.89 (0.87-0.91) | 0.89 (0.87-0.91) | 0.89 (0.87-0.91) |

Statin Continuer, receipt of 2 or more prescriptions, first prescription > 1 year before index date and last prescription within 6 months

before index date; Statin Discontinuer, receipt of 1 prescription > 1 year before index date.

^a^ Adjusted for obesity (BMI ≥30 kg/m^2^), ever smoking, chronic use of aspirin/NSAIDs (>5 years), hormone replacement therapy, alcohol

consumption, diabetes mellitus, performance of bowel screening, non-statin cholesterol lowering medication, and pre-treatment cholesterol.

^b^ Adjusted for variables in original model, including BMI as continuous. Linear regression imputed missing BMI. Standard errors

were adjusted to account for variability among the imputations.

^c^ Adjusted for variables in original model, excluding subjects with prior history of lung, prostate, or breast cancers (n=3,684)

^d^ Per 1 unit (mmol/L) increase in serum total cholesterol.
