# Supplementary material for: Disentangling the Association between Statins, Cholesterol, and Colorectal Cancer: A Nested Case-Control Study
Source: PLoS Med. 2016 Apr 26;13(4):e1002007. doi: 10.1371/journal.pmed.1002007 (PMC4846028; doi:10.1371/journal.pmed.1002007)
Supplement: S2 Table — (DOCX) [file pmed.1002007.s004.docx]

| S2 Table. ORs for colorectal cancer risk by change in serum total cholesterol, excluding other prior cancers | | | | | | |
| --- | --- | --- | --- | --- | --- | --- |
| Model | Cases^a^ | | Controls^a^ | OR (95% CI) for no change or increase^b^ | Original  OR (95% CI) per 1 mmol/L decrease | Excluding prior cancers  OR (95% CI) per 1 mmol/L decrease |
| **Statin non users** | | | |  |  |  |
| Adjusted^c^ | | 1,752 | 2,455 | 1.00 | 1.47 (1.35-1.60) | 1.47 (1.34-1.60) |
| Most fully adjusted^d^ | | 1,084 | 1,449 | 1.00 | 1.49 (1.32-1.69) | 1.49 (1.31-1.70) |
| **Statin users** | |  |  |  |  |  |
| Adjusted^c^ | | 2,981 | 5,087 | 1.00 | 1.09 (1.04-1.13) | 1.09 (1.04-1.13) |
| Most fully adjusted^d^ | | 2,341 | 3,845 | 1.00 | 1.23 (1.15-1.32) | 1.22 (1.14-1.31) |

^a^ Limited to cases and controls with at least 2 total cholesterol measurements, separated by at least 1 year, with the last measurement occurring at least 1 year before the index date of colorectal cancer diagnosis, and without history of prior lung, prostate, or breast cancer.

^b^ Reference group includes subjects with no change or increase in total cholesterol between the first and last total cholesterol measurement recorded

^c^ Adjusted for age, sex duration of follow-up, calendar period, obesity (BMI ≥30 kg/m^2^), ever smoking, chronic use of aspirin or non-steroidal anti-inflammatory medications, hormone replacement therapy, alcohol consumption, diabetes mellitus, and performance of bowel screening

^d^ Adjusted for variables in adjusted model, as well as non-statin cholesterol lowering medication, weight loss during follow-up, and first available total cholesterol measurement during follow-up
