# Supplementary material for: Disentangling the Association between Statins, Cholesterol, and Colorectal Cancer: A Nested Case-Control Study
Source: PLoS Med. 2016 Apr 26;13(4):e1002007. doi: 10.1371/journal.pmed.1002007 (PMC4846028; doi:10.1371/journal.pmed.1002007)
Supplement: S3 Table — (DOCX) [file pmed.1002007.s005.docx]

| S3 Table. ORs for association between CRC and cholesterol measured at different time intervals before diagnosis among:  Statin non users (n=15,052 cases; n=46,043 controls)   \|  \|  \| Total cholesterol measurement window \| \| \| \| \| \| --- \| --- \| --- \| --- \| --- \| --- \| --- \| \|  \|  \| < 6 months \|  \| 6 to 12 months \| 12 to 24 months \| > 24 months \| \| **Total Cholesterol Level^a^** \|  \| Adjusted^b^ OR  (95% CI) \|  \| Adjusted^b^ OR  (95% CI) \| Adjusted^b^ OR  (95% CI) \| Adjusted^b^ OR  (95% CI) \| \| <4 mmol/L \| Reference \| \| \| Reference \| Reference \| Reference \| \| 4-5 mmol/L \| 0.46 (0.31-0.68) \| \| \| 0.70 (0.41-1.21) \| 1.01 (0.74-1.40) \| 1.12 (0.89-1.41) \| \| 5-6 mmol/L \| 0.28 (0.19-0.43) \| \| \| 0.57 (0.33-0.97) \| 0.92 (0.67-1.28) \| 0.98 (0.78-1.24) \| \| 6-7 mmol/L \| 0.31 (0.20-0.48) \| \| \| 0.54 (0.30-0.97) \| 0.87 (0.61-1.23) \| 0.99 (0.78-1.26) \| \| >7 mmol/L \| 0.26 (0.15-0.45) \| \| \| 0.28 (0.13-0.60) \| 0.72 (0.47-1.10) \| 1.03 (0.77-1.38) \| \| Continuous^c^ \| 0.76 (0.68-0.84) \| \| \| 0.79 (0.69-0.90) \| 0.92 (0.85-0.99) \| 0.98 (0.93-1.03) \|   Statin users (n=5,100 cases; n=9,704 controls) | | | | |
| --- | --- | --- | --- | --- | --- | --- | --- | --- | --- | --- | --- | --- | --- | --- | --- | --- | --- | --- | --- | --- | --- | --- | --- | --- | --- | --- | --- | --- | --- | --- | --- | --- | --- | --- | --- | --- | --- | --- | --- | --- | --- | --- | --- | --- | --- | --- | --- | --- | --- | --- | --- | --- | --- | --- | --- | --- | --- | --- | --- | --- | --- | --- | --- | --- | --- | --- | --- |
| <4 mmol/L | Reference | Reference | Reference | Reference |
| 4-5 mmol/L | 0.60 (0.52-0.70) | 0.88 (0.76-1.03) | 0.90 (0.81-0.99) | 0.95 (0.86-1.05) |
| 5-6 mmol/L | 0.54 (0.44-0.66) | 0.78 (0.64-0.94) | 0.83 (0.73-0.95) | 1.06 (0.94-1.19) |
| 6-7 mmol/L | 0.52 (0.38-0.71) | 0.70 (0.53-0.92) | 0.86 (0.71-1.04) | 0.96 (0.82-1.12) |
| >7 mmol/L | 0.59 (0.38-0.43) | 0.77 (0.51-1.17) | 0.80 (0.61-1.06) | 0.97 (0.79-1.20) |
| Continuous^c^ | 0.79 (0.73-0.84) | 0.91 (0.85-0.91) | 0.95 (0.91-0.99) | 1.00 (0.96-1.04) |

Statin users and nonusers, adjusted for statin use (n=22,163 cases, n=86,538 controls)

| <4 mmol/L | Reference | Reference | Reference | Reference |
| --- | --- | --- | --- | --- |
| 4-5 mmol/L | 0.67 (0.61-0.75) | 0.87 (0.78-0.98) | 0.92 (0.85-0.99) | 0.98 (0.91-1.06) |
| 5-6 mmol/L | 0.50 (0.44-0.57) | 0.77 (0.67-0.88) | 0.84 (0.77-0.93) | 0.98 (0.90-1.06) |
| 6-7 mmol/L | 0.44 (0.38-0.52) | 0.66 (0.56-0.79) | 0.81 (0.71-0.91) | 0.99 (0.90-1.09) |
| >7 mmol/L | 0.44 (0.34-0.56) | 0.53 (0.41-0.68) | 0.73 (0.61-0.86) | 0.93 (0.82-1.06) |
| Continuous^c^ | 0.77 (0.74-0.80) | 0.86 (0.83-0.90) | 0.93 (0.90-0.96) | 0.99 (0.97-1.01) |

^a^ Last total cholesterol value measured in each specified time window prior to the index date of colorectal cancer diagnosis

^b^ Adjusted for obesity (BMI ≥30 kg/m^2^), ever smoking, chronic use of aspirin or NSAIDs, hormone replacement therapy, alcohol consumption, diabetes mellitus, performance of bowel screening, and use of non-statin cholesterol lowering medication.

^c^ Per 1 unit (mmol/L) increase in serum total cholesterol
