# Supplementary material for: Disentangling the Association between Statins, Cholesterol, and Colorectal Cancer: A Nested Case-Control Study
Source: PLoS Med. 2016 Apr 26;13(4):e1002007. doi: 10.1371/journal.pmed.1002007 (PMC4846028; doi:10.1371/journal.pmed.1002007)
Supplement: S4 Table — (DOCX) [file pmed.1002007.s006.docx]

| S4 Table. ORs for colorectal cancer risk by change in triglycerides, LDL, and HDL cholesterol | | | | | |  |
| --- | --- | --- | --- | --- | --- | --- |
| Model^a^ |  | OR (95% CI) per 1 mmol/L decrease in lipid concentration | | | | |
|  | Reference^b^ | Total Cholesterol | Triglycerides | LDL-Cholesterol | HDL-Cholesterol | |
| **Statin non users** |  |  |  |  |  | |
| Most fully adjusted^c^ | 1.00 | 1.49 (1.32-1.69) | 1.16 (1.06-1.27) | 1.37 (1.11-1.69) | 2.14 (1.49-3.07) | |
| **Statin users** |  |  |  |  |  | |
| Most fully adjusted^c^ | 1.00 | 1.23 (1.15-1.32) | 1.12 (0.94-1.34) | 1.21 (1.10-1.34) | 1.31 (1.07-1.60) | |

^a^ Limited to cases and controls with at least 2 total cholesterol measurements, separated by at least 1 year, with the last measurement occurring at least 1 year before the index date of colorectal cancer diagnosis.

^b^ Reference group includes subjects with no change or increase in total cholesterol between the first and last total cholesterol measurement recorded

^c^ Adjusted for age, sex duration of follow-up, calendar period, obesity (BMI ≥30 kg/m^2^), ever smoking, chronic use of aspirin or non-steroidal anti-inflammatory medications, hormone replacement therapy, alcohol consumption, diabetes mellitus, performance of bowel screening, non-statin cholesterol lowering medication, weight loss during follow-up, and first available lipid measurement during follow-up
