# Supplementary material for: Disentangling the Association between Statins, Cholesterol, and Colorectal Cancer: A Nested Case-Control Study
Source: PLoS Med. 2016 Apr 26;13(4):e1002007. doi: 10.1371/journal.pmed.1002007 (PMC4846028; doi:10.1371/journal.pmed.1002007)
Supplement: S1 Text — (DOCX) [file pmed.1002007.s007.docx]

S1 Text. THIN code list for colorectal cancer, statin drugs, and lipid profile

**Colorectal cancer (READ codes):**

**Non-site specific codes:**

B13..00; B13z.11; B13z.00; B13y.00; B803z00; B803.00

**Ascending colon:**

B136.00; B130.00; B134.00; B134.11; B803000; B803400; B803600; B135.00; B803500

**Transverse colon:**

B131.00; B803100

**Descending colon:**

B803200; B137.00; B132.00; B803700

**Recto-sigmoid:**

B133.00; B803300; B804z00; B804.00; B804000; B14z.00; B14..00; B140.00; B141.12; B141.00; B804100; B141.11

**Statins (DRUG codes):**

83030998; 83099998; 86020998; 86467998; 8648998; 86787998; 86788998; 86789998; 86791998; 86794998; 86795998; 86796998; 86797998; 86798998; 87373998; 87418998; 87916998; 87917998; 87918998; 88534998; 89153996; 89153997; 89153998; 89154996; 89154997; 89154998; 89306996; 89306997; 89306998; 89311996; 89311997; 89311998; 90309998; 90310998; 90973998; 91194998; 92220998; 92408998; 92409998; 92410998; 92447998; 92448997; 92448998; 92471998; 92539998; 92804996; 92804997; 92804998; 92805997; 92805998; 93243996; 93243997; 93243998; 93244996; 93244997; 93244998; 93619996; 93619997; 93619998; 93620996; 93620997; 93620998; 94830990; 94849990; 94850990; 95277990; 95278990; 95405990; 95442990; 95449990; 95450990; 95451990; 95471990; 95472990; 95474990; 95475990; 95478990; 95479990; 95480990; 95481990; 95482990; 95483990; 95486990; 95487990; 95493990; 95494990; 95495990; 95500990; 95501990; 95502990; 95508990; 95549990; 95550990; 95551990.

**Lipid profile (AHD codes):**

**Total cholesterol:** 1001400017

**LDL:** 1001400035

**HDL:** 1001400031

**Triglycerides:** 1001400045
