# Supplementary material for: Disentangling the Association between Statins, Cholesterol, and Colorectal Cancer: A Nested Case-Control Study
Source: PLoS Med. 2016 Apr 26;13(4):e1002007. doi: 10.1371/journal.pmed.1002007 (PMC4846028; doi:10.1371/journal.pmed.1002007)
Supplement: S2 Text — (PDF) [file pmed.1002007.s008.pdf]

STROBE Statement—Checklist of items that should be included in reports of ***case-control studies***

	Item No	Recommendation	p=paragraph #
**Title and abstract**	1	(*a*) Indicate the study's design with a commonly used term in the title or the abstract	YES
		(*b*) Provide in the abstract an informative and balanced summary of what was done and what was found	YES
**Introduction**			
Background/rationale	2	Explain the scientific background and rationale for the investigation being reported	p2-4
Objectives	3	State specific objectives, including any prespecified hypotheses	
**Methods**			
Study design	4	Present key elements of study design early in the paper	p2
Setting	5	Describe the setting, locations, and relevant dates, including periods of recruitment, exposure, follow-up, and data collection	p1-6
Participants	6	(*a*) Give the eligibility criteria, and the sources and methods of case ascertainment and control selection. Give the rationale for the choice of cases and controls	p3-4
		(*b*) For matched studies, give matching criteria and the number of controls per case	
Variables	7	Clearly define all outcomes, exposures, predictors, potential confounders, and effect modifiers. Give diagnostic criteria, if applicable	p3-6
Data sources/ measurement	8*	For each variable of interest, give sources of data and details of methods of assessment (measurement). Describe comparability of assessment methods if there is more than one group	p1
Bias	9	Describe any efforts to address potential sources of bias	p7-9
Study size	10	Explain how the study size was arrived at	
Quantitative variables	11	Explain how quantitative variables were handled in the analyses. If applicable, describe which groupings were chosen and why	p7-9
Statistical methods	12	(*a*) Describe all statistical methods, including those used to control for confounding	p7-9
		(*b*) Describe any methods used to examine subgroups and interactions	
		(*c*) Explain how missing data were addressed	Results, p5
		(*d*) If applicable, explain how matching of cases and controls was addressed	p4
		(*e*) Describe any sensitivity analyses	Results, p5
**Results**			
Participants	13*	(a) Report numbers of individuals at each stage of study—eg numbers potentially eligible, examined for eligibility, confirmed eligible, included in the study, completing follow-up, and analysed	p1, Table 1
		(b) Give reasons for non-participation at each stage	
		(c) Consider use of a flow diagram	
Descriptive data	14*	(a) Give characteristics of study participants (eg demographic, clinical, social) and information on exposures and potential confounders	p1, Table 1
		(b) Indicate number of participants with missing data for each variable of interest	
Outcome data	15*	Report numbers in each exposure category, or summary measures of exposure	p1-5
Main results	16	(*a*) Give unadjusted estimates and, if applicable, confounder-adjusted estimates and their precision (eg, 95% confidence interval). Make clear which confounders were adjusted for and why they were included	p1-5, Tables 2-4, Fig 1
		(*b*) Report category boundaries when continuous variables were categorized	
		(*c*) If relevant, consider translating estimates of relative risk into absolute risk for a meaningful time period	N/A

Other analyses	17	Report other analyses done—eg analyses of subgroups and interactions, and sensitivity analyses	p5 S1-4
**Discussion**			
Key results	18	Summarise key results with reference to study objectives	p1
Limitations	19	Discuss limitations of the study, taking into account sources of potential bias or imprecision. Discuss both direction and magnitude of any potential bias	p7-9
Interpretation	20	Give a cautious overall interpretation of results considering objectives, limitations, multiplicity of analyses, results from similar studies, and other relevant evidence	p1-10
Generalisability	21	Discuss the generalisability (external validity) of the study results	p9
**Other information**			
Funding	22	Give the source of funding and the role of the funders for the present study and, if applicable, for the original study on which the present article is based	Included

*Give information separately for cases and controls.

Note: An Explanation and Elaboration article discusses each checklist item and gives methodological background and published examples of transparent reporting. The STROBE checklist is best used in conjunction with this article (freely available on the Web sites of PLoS Medicine at http://www.plosmedicine.org/, Annals of Internal Medicine at http://www.annals.org/, and Epidemiology at http://www.epidem.com/). Information on the STROBE Initiative is available at http://www.strobe-statement.org.
